# Cases in Electro-Therapeutics

**Published:** 1871-02

**Authors:** Wm. J. Maynard

**Affiliations:** Chicago


ARTICLE VI.—*Cases in Electro-Therapeutics. Reported by* WM. J. MAYNARD, M.D., Chicago.

Earnest and well directed study has already demonstrated the fact that Electricity holds a high place in the treatment of disease, and it is truly encouraging to see what progress has been made in it, especially in the last few years. Besides the particular disorders of the nervous system, to which it has usually been applicable, it is making rapid strides in other departments of medicine. In giving this subject particular study of late, it has been my good fortune to have seen some remarkable cures made, and from my own experience I present the three following cases, thinking they might not prove uninteresting to the readers of the JOURNAL.

Case I. Mr. R. was sent to me by a leading medical gentleman of the city to see what electrical treatment would do for him, as all other remedies had failed to give the required benefit. The patient had been confined to his bed with typhoid fever, and during convalescence found that he had a partial paralysis of the left leg, with great anasarca. When I first saw him he was scarcely able to walk, but before six applications of the battery had been made he was able to walk several blocks comfortably, and with great diminution in the size of the leg. After twelve applications the patient was dismissed perfectly cured.

Case 2. At the request of the same physician, I was called to see Mr. D. Upon examination I found the left leg enormously distended by anasarca, great numbness of the entire member, with inactivity of the circulation. My success with the first led me to adopt the same mode of treatment, and although the pathological condition was entirely different, it was surprising with what rapidity the leg regained its normal condition after each application of the battery. After eleven sittings the patient was able to walk and attend to his business.

In these two cases, I do not attempt to deny that the general course of treatment at first adopted would, perhaps, after a lapse of time, have effected a cure, but I do claim that a far more speedy resolution was brought about by the electric agent. The great absorbing and alterative effect produced by the galvanic current is better illustrated by the following case, which it has been our pleasure to witness:

Mr. T., of this city, consulted me for the purpose of getting rid of a tumor, a little larger, perhaps, than a hen's egg, situated on the right side of the neck, along the anterior border of the sterno-cleido muscle. The tumor had been growing for a long time and seemed to be slowly increasing. The diagnosis that I made, *i. e.*, that it was an enlargement of the lymphatic gland, he said, was confirmed by the opinion of other physicians. As he had too great a horror of the surgeon's knife to yield to the general mode of extirpating these tumors, I must confess that I entered upon the electric treatment with not much confidence in the result. I

used a rather powerful induction current, and passed it transversely through the substance of the tumor. After a few applications, each sitting lasting about twenty minutes, there was a perceptible diminution in the size of the gland. With this encouragement, the treatment was continued until sixteen applications were made, when all that remained was a small fluctuating mass, which I punctured, and drew out some fluid which had every appearance of lymph. This continued to discharge for some time, but the patient is now apparently perfectly well.

